# 2883. Comparative outcomes following Candida bloodstream infections in patients with left ventricular assist devices (LVAD)

**DOI:** 10.1093/ofid/ofad500.160

**Published:** 2023-11-27

**Authors:** Armaghan-e-Rehman Mansoor, Gayathri Krishnan, Julio C Zuniga-Moya, Dorothy Sinclair, Andrej Spec, Ige George

**Affiliations:** Washington University in St. Louis, Lexington, KY; Washington University School of Medicine, St. Louis, Missouri; Washington University School of Medicine in St. Louis, St. Louis, Missouri; Washington University School of Medicine in St. Louis, St. Louis, Missouri; Washington University in St. Louis, Lexington, KY; Washington University, St. Louis, MO

## Abstract

**Background:**

Left ventricular assist devices (LVAD) are an important management strategy for end-stage heart failure, however they carry a risk of infection. This study examines attributable outcomes of LVAD presence in patients presenting with *Candida* bloodstream infection (Candidemia).

**Methods:**

1233 patients with candidemia admitted to Barnes Jewish Hospital between January 2010-December 2021 were retrospectively included, of whom 39 had an LVAD at the time of infection. Demographics, comorbidities, microbiologic data, and risk factors for infection were analyzed. Outcome measures included 30 and 90-day mortality, need for mechanical ventilation, and dialysis. Risk factors for candidemia, and outcomes following infection were compared between patients with and without LVAD presence.

**Results:**

Candidemic patients in the LVAD cohort were more likely to be male (77%) compared to the non-LVAD group (54%) (Table 1). Patients with an LVAD were more likely to have central venous access (p-value 0.04), and to have received extracorporeal membrane oxygenation (ECMO) prior to infection (67% vs 46%, p-value=0.01). There were no statistically significant differences in rates of parenteral nutrition, abdominal surgeries, liver disease or malignancies between the two cohorts. *C. parapsilosis* was the most common causative species in patients with LVAD (38% in LVAD group vs 16%) followed by *C. albicans* compared to the non-LVAD cohort (24% in LVAD group vs 39%). Patients in both groups had similar rates of mechanical ventilation, and hemodialysis need. HeartMate2 was the most common LVAD strategy in 21 patients (54%). Two patients required LVAD exchange as part of treatment.

Presence of an LVAD did not significantly impact 30-day (31% in LVAD group vs 35%, p-value=0.62) or 90-day mortality (38% in LVAD group vs 41%, p-value 0.77). Within the LVAD cohort, 90-day mortality was associated with a shorter time duration from LVAD placement to candidemia.

**Figure:**
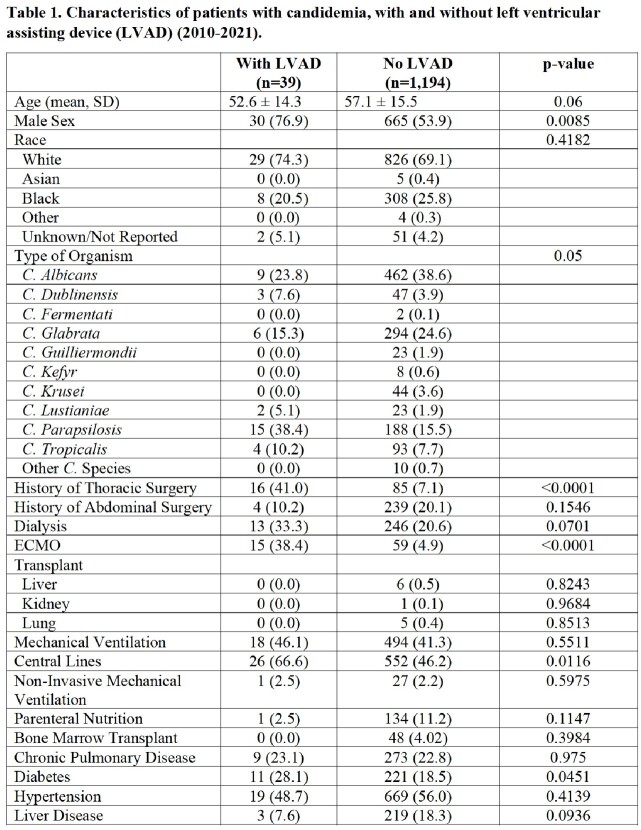

**Conclusion:**

In patients presenting with Candidemia, presence of an LVAD did not impact 30 or 90-day mortality. A shorter time from LVAD placement to infection was associated with poorer outcomes.

**Disclosures:**

**All Authors**: No reported disclosures

